# Interfacial Properties of MoS_2_ Thin Films Grown on Functional Substrates

**DOI:** 10.1021/acsomega.6c03932

**Published:** 2026-09-07

**Authors:** Hafiz Sami Ur Rehman, Nunzia Coppola, Alice Galdi, Sandeep Kumar Chaluvadi, Shyni Punathum Chalil, Pasquale Orgiani, Sara Passuti, Regina Ciancio, Paolo Barone, Luigi Maritato, Carmela Aruta

**Affiliations:** † Dipartimento di Ingegneria Industriale-DIIN, 19028 Università Degli Studi di Salerno, Fisciano, Salerno 84084, Italy; ‡ 518735 CNR-IOM, Strada Statale 14 Km 163.5, Basovizza, Trieste 34149, Italy; § 18469 Area Science Park, Padriciano 99, Trieste 34149, Italy; ∥ 9327 CNR-SPIN, via del Fosso del Cavaliere 100, Roma 00133, Italy

## Abstract

Interface chemistry and defect formation in MoS_2_ thin films grown on single crystal substrates critically determine the electronic structure of MoS_2_ and thus can strongly modify material functionality relevant for many applications, including electronics, optoelectronics, and energy-related catalysis. We investigate MoS_2_ grown on three technologically relevant substrates, namely SrTiO_3_(111), *c*-axis Al_2_O_3_(0001) and 6H-SiC(0001). Experimental investigations by temperature dependent resistivity, photoemission spectroscopy and scanning transmission electron microscopy with coupled energy dispersive spectroscopy, with the support of theoretical calculation by Density Functional Theory, allow the identification of the substrate induced specific defects and their correlation with the electronic properties. Ti interdiffusion in SrTiO_3_/MoS_2_ generates p-type states near the Fermi level, leading to metallic transport. Al_2_O_3_/MoS_2_ exhibits a high density of sulfur-related defects that introduce localized states and yield nearly temperature independent conductivity. SiC/MoS_2_ exhibits significant interface disorder resulting in a semiconducting temperature dependent resistivity, yet deviating from the ideal bulk-like behavior. These results demonstrate how substrate choice governs defect formation and ultimately dominates the electronic behavior of MoS_2_ thin films, making the control of film/substrate interactions essential for the engineering of new functional devices.

## Graphical abstract

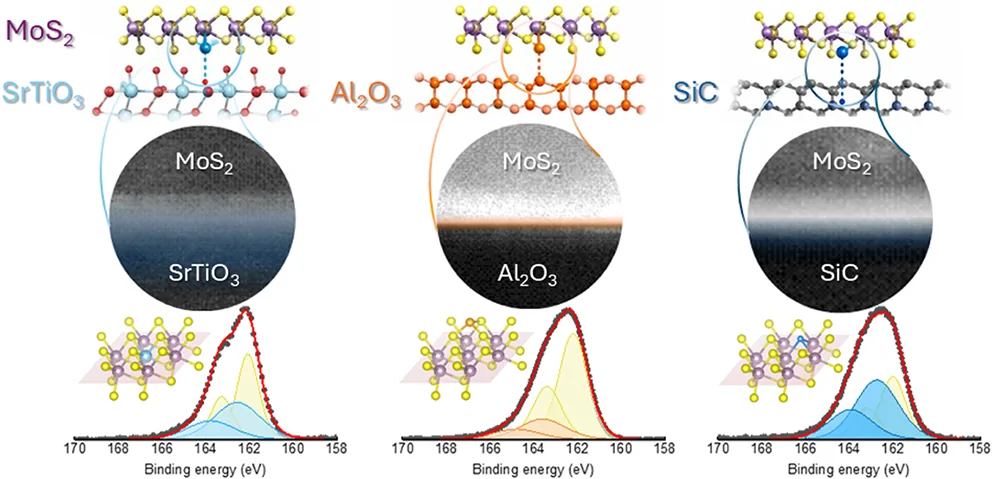

## 1 Introduction

Among the various transition-metal dichalcogenides (TMDCs), MoS_2_ has gained increasing attention for its distinctive fundamental properties and technological relevance across a broad range of electronic, optoelectronic, quantum-technology, and energy-conversion applications.^1–5^

When MoS_2_ is grown as a thin film on a specific substrate, its structural, electronic, and optical responses become sensitive to the interface between film and substrate.^6–10^ Defect engineering and interface control, especially on oxides and polar substrates, have been largely used to tune the functional properties of MoS_2_.^11–16^ Sulfur related defects or other point defects, such oxygen or ion interdiffusion from the substrate, can create localized states within the bandgap, modify the charge density and enhance catalytic activity.^17–19^ In emerging quantum-technology applications based on MoS_2_, substrate-induced defects can strongly influence spin—valley excitonics, a key ingredient for exciton—based quantum platforms.^20–22^ In the ultrathin limit, the interface becomes extremely relevant, introducing chemical defects, structural disorder, new electronic and excitonic features, thus making interface engineering a powerful means to modulate their formation and, possibly, giving rise to new states not present in bulk MoS_2_.^23,24^ Different interfaces between film and substrate can induce specific modifications having direct relevance for planar device architectures that integrate MoS_2_ on insulating or semiconducting substrates.^23^

In this work, we investigate polycrystalline MoS_2_ films grown by Pulsed Laser Deposition (PLD) on SrTiO_3_(111) (STO), c-axis Al_2_O_3_(0001) and 6H-SiC (0001)­substrates, which are commonly used for device fabrication in semiconductor and oxide-electronics. Polycrystalline morphology appears regularly in MoS_2_ thin films, grown by chemical (CVD) or physical (magnetron and PLD) vapor deposition techniques.^25–28^ Literature results suggest that granularity enables band-gap tuning through quantum confinement, which improves optoelectronics and photocatalytic performance.^26,29,30^ In addition, it has been reported that phonon-boundary scattering reduces thermal conductivity in thermoelectric devices^31,32^ and polycrystallinity facilitates defect-driven switching in memristive devices.^33,34^ Despite domain boundaries, polycrystalline MoS_2_ can show transport behavior comparable to single crystals and is suitable for large-area and flexible electronics.^35,36^ For hydrogen evolution reaction (HER), polycrystallinity increases active sites and their efficacy.^37^

The STO, Al_2_O_3_ and SiC substrates used in this work are all wide-band-gap materials, thus providing excellent platforms for MoS_2_ based electronic and optoelectronic devices. STO provides a high-k dielectric response which allows efficient charge carrier modulation in field-effect transistors.^12,38^ The formation of a covalent-like interaction can lead to charge transfer between the substrate and the MoS_2_ layer.^11,39,40^ Al_2_O_3_, with its chemical stability, is a common substrate for growing MoS_2_ films and is widely used as a high-k gate dielectric in MoS_2_ transistors. Several reports have highlighted that dipole–dipole interactions existing between S and Al atoms allow a good alignment of the MoS_2_ layers.^41,42^ In contrast, despite the promising potential of MoS_2_ on SiC, the characteristics achieved so far has not met the requirements of advanced electronic devices.^43^ However, given the high thermal conductivity, large breakdown field, and excellent radiation stability of SiC, together with the theoretically demonstrated charge transfer at the interface, the SiC/MoS_2_ interface provides significant potential for exploring new functionalities.^44^

We employ PLD to grow the MoS_2_ films, as we have previously reported for MoS_2_ with different compositions.^15,45,46^ Although less explored than other conventional growth methods for TMDCs, PLD has shown strong potential for the controlled synthesis of high-quality MoS_2_.^27,28,47^ The flexibility and scalability of PLD allows engineering interfaces and heterostructures, and optimizing film quality for next-generation TMDC-based devices.

Our aim is to uncover how substrate interactions and defect profiles combine to modify the electronic landscape of MoS_2_. To address these aspects, we combine X-ray photoemission spectroscopy (XPS) and high-resolution transmission electron microscopy (HRTEM) coupled with Energy-Dispersive Spectroscopy (STEM-EDS) together with Density Functional Theory (DFT) calculations, to identify the substrate-induced defects in thin films of MoS_2_ grown on the three selected substrates and correlate them with the temperature dependent resistivity behavior observed in thicker films. Our findings highlight how substrate selection governs defect formation and interfacial chemistry, ultimately shaping the electronic structure and transport properties of MoS_2_ thin films. Understanding these mechanisms is a critical step toward the rational design of next-generation planar devices and catalytic systems that rely on few-layer MoS_2_ with engineered optical, electronic, and chemical properties.^45,46^

## 2 Results and Discussion

Temperature-dependent electrical transport measurements on MoS_2_ films 60–80 nm thick, all grown under the same conditions but on different substrates, STO, Al_2_O_3_ and SiC, are reported in Figure 1. It can be observed that markedly different behaviors occur depending on the substrate. STO/MoS_2_ exhibits good conductive behavior, with a very low room-temperature resistivity of 0.22 mΩ·cm. In contrast, SiC/MoS_2_ shows a more resistive semiconducting behavior, with a slightly wavy temperature dependence and a higher room-temperature resistivity of 3.8 mΩ·cm. Al_2_O_3_/MoS_2_ displays an intermediate trend, essentially semiconducting, but with the highest room-temperature resistivity among the three, reaching 7.3 mΩ·cm. The three temperature-dependent transport curves indicate that the electronic behavior of MoS_2_ is strongly influenced by the underlying substrate. The metallic-like response observed for STO/MoS_2_ suggests that this interface may promote efficient charge transfer, possibly through favorable interfacial electronic coupling or substrate-induced modification of the electronic structure. The highly resistive, slightly wavy semiconducting behavior of SiC/MoS_2_ is indicative of interface-induced states that weakly modulate the activation behavior. Al_2_O_3_/MoS_2_ shows an intermediate, essentially semiconducting trend, which could arise from moderate interface disorder that limits the carrier mobility.

**Figure 1:**
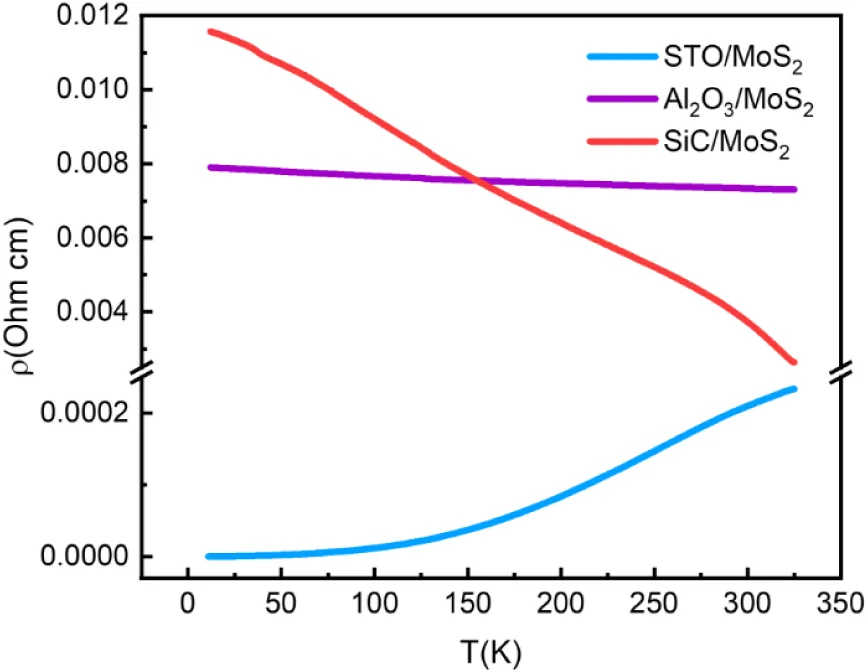
Resistivity measurements as a function of temperature of MoS_2_films grown on STO, Al_2_O_3_ and SiC substrates.

Overall, the distinct behaviors observed for the three substrates are consistent with different interfacial interactions. Previous research on similar samples demonstrated that substrate-induced strain is not sufficient to explain the structural results obtained by X-ray diffraction (XRD).^15^ Therefore, to clarify the origin of these interface related phenomena, we performed XPS measurements by synchrotron radiation on ultrathin films only 2 ML thick, a thickness specifically chosen to enhance interface response. The XPS experiments were carried out *in situ* by transferring the samples directly from the PLD to the analysis chamber without air exposure, a crucial aspect given the extreme sensitivity of 2 monolayers (ML) films to ambient contamination. The XPS core level measurements of Mo 3d and S 2p are shown in Figure 2a and b, respectively. It can be observed that the shape of the Mo 3d signal does not change significantly across the MoS_2_ films grown on the three different substrates, except for the peak broadening, which increases from STO to Al_2_O_3_ to SiC and thus indicates a higher degree of disorder and the presence of additional components associated with defect states or chemical environments, as demonstrated by the Mo 3d fit reported in the Figure S1a of the Supporting Information. The thickness dependent study on STO/MoS_2_ system reported in Figure S1b demonstrates that the differences among the MoS_2_ films can be very likely associated with the interface between film and substrate. However, more pronounced modifications can be observed in the profile of the S 2p core levels, where all spectra exhibit a clear asymmetry toward the higher BE region. In the STO/MoS_2_ interface, the spectrum is relatively sharp and well-defined. Moving to Al_2_O_3_/MoS_2_ the peaks exhibit slight broadening which becomes most pronounced in the SiC/MoS_2_ sample. These variations reflect substrate-induced modifications in the sulfur chemical states and point to a progressive increase in defect density from STO to Al_2_O_3_ to SiC. We therefore performed a fit of S 2p spectra, to highlight the main differences in the shape of the S 2p core levels, shown in Figure 3. Given the overall XPS energy resolution of about 300 meV, closely spaced defect related chemical shifts cannot be uniquely disentangled. The assignment of specific interfacial configurations is therefore based on the combined consistency among XPS, STEM-EDS, valence band spectra and DFT calculations, as further substantiated in the following sections. In this context, the fit is performed with two 2p_3/2_ – 2p_1/2_ doublets, as detailed in the Supporting Information. Doublet A corresponds to stoichiometric sulfur and appears at almost the same BE for all samples. Doublet B is left free to vary and contains the main differences among the three samples.

**Figure 2:**
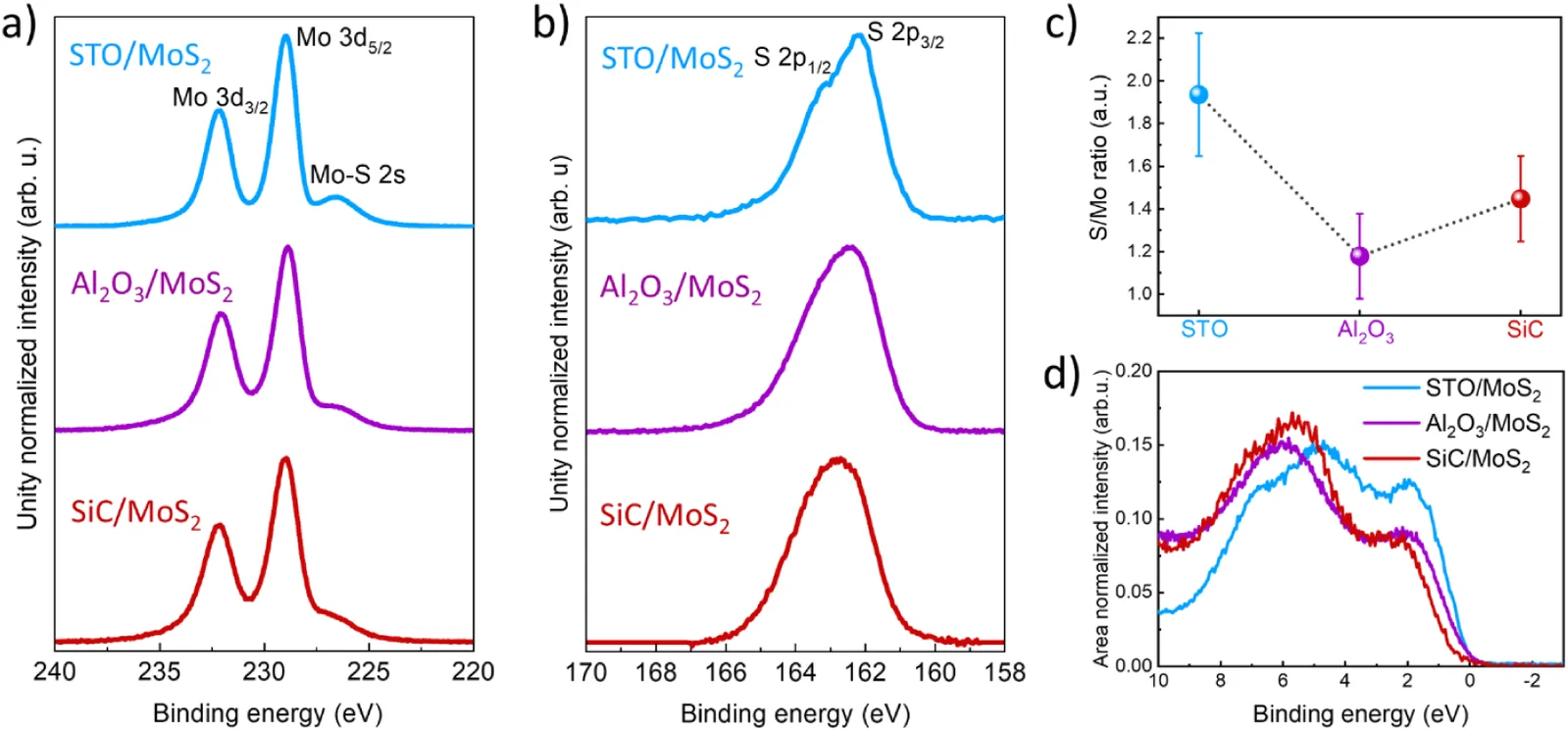
XPS measurements on 2 ML MoS_2_ films grown on STO, Al_2_O_3_ and SiC. a) Mo 3d core level experimental spectra; b) S 2p core level experimental spectra; c) Mo/S ratio obtained from a) and b) measurements; d) VB measurements normalized to the area.

**Figure 3:**
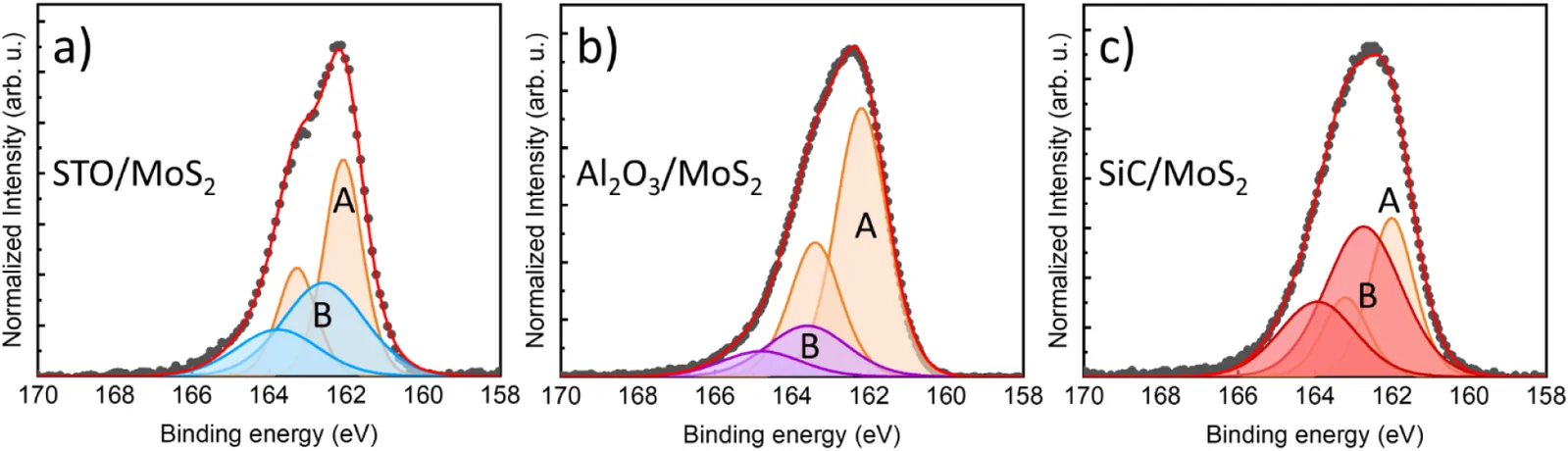
Two components fit of 2 ML thick MoS_2_ films on a) STO, b) Al_2_O_3_ and c) SiC substrates.

The calculation of the S/Mo ratio derived from the S 2p and Mo 3d core levels in the XPS spectra, is reported in Figure 2c. All three samples contain sulfur vacancies, but the one grown on STO is the closest to stoichiometric 2 value, with S/Mo=1.94. The sample grown on Al_2_O_3_ shows the lowest ratio S/Mo=1.18, while the SiC/MoS_2_ sample displays an intermediate value of S/Mo=1.45. The decreasing trend of the Mo 3d—S 2s related feature in Figure 2a is consistent with the reduction of the S/Mo ratio.

In Figure 2d the VB measurements show that, compared to the MoS_2_ on SiC, there is an increase in Mo 3d states near the Fermi level (E_F_) in the MoS_2_ on Al_2_O_3_ sample and an even stronger increase in the case of STO substrate. The calculated valence band maximum (VBM) values result +0.420 eV (SiC/MoS_2_), +0.026 eV (Al_2_O_3_/MoS_2_) and +0.020 eV (STO/MoS_2_), as reported in Supporting Information. The shift of the VBM closer to E_F_ from SiC to Al_2_O_3_ to STO is consistent with the improved conduction observed for Al_2_O_3_ and STO compared to SiC (Figure 1). However, a better understanding of defect-related and interfacial modifications is needed to clarify the origin of these findings.

To probe interfacial chemical disorder and elemental interdiffusion which can be at the origin of defect-induced electronic states, STEM-EDS mapping was performed across the film/substrate interface. Although the XPS results presented in this work refer to ultrathin films, the STEM investigations were carried out on thicker films grown under identical conditions. The use of thicker samples allowed the preparation of extended and more homogeneous cross-sectional regions during FIB-SEM specimen preparation, facilitating a more reliable structural and compositional analysis of the film/substrate interface. Cross-sectional lamellae were extracted by FIB-SEM to expose the film/substrate interface in cross-sectional geometry, allowing direct investigation of the interfacial region. STEM-EDS mapping was subsequently performed, acquiring elemental line scans across the interface together with spatially resolved chemical maps.

Concerning the sample deposited on STO, from the map overlays, we can observe a constant interdiffusion of Ti into the MoS_2_ thin film across the length of the sample, of about 5 nm depth (Figure 4). Although STEM-EDS elemental mapping has intrinsic limitations in resolving weak elemental signals at interfaces, the STO/MoS_2_ sample clearly exhibits a pronounced Ti enrichment extending from the substrate into the interfacial region of the MoS_2_ film. This observation is consistent with Ti interdiffusion at the interface.

**Figure 4:**
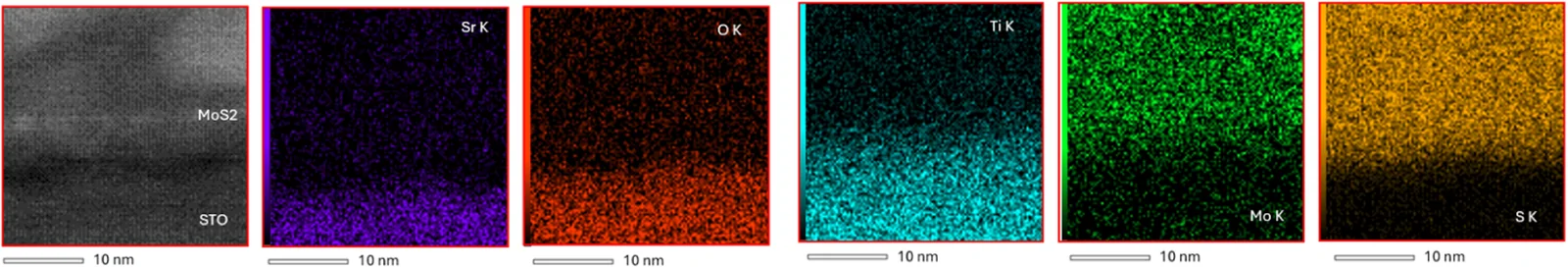
Cross-sectional HAADF-STEM image of the STO/MoS_2_ interface (leftmost panel), together with corresponding STEM-EDS elemental maps acquired from the same region. The chemical maps display the spatial distribution of Sr K, O K, Ti K, Mo K, S K signals, respectively.

The films deposited on Al_2_O_3_(Figure 5) and SiC (Figure 6) result as being more stable. No interdiffusion is detected at the interface. However, a layer containing oxygen in the MoS_2_ sample deposited on SiC of about 16 nm, which is observed across the whole film length, suggests oxidation of the substrate surface prior to the film deposition.

**Figure 5:**
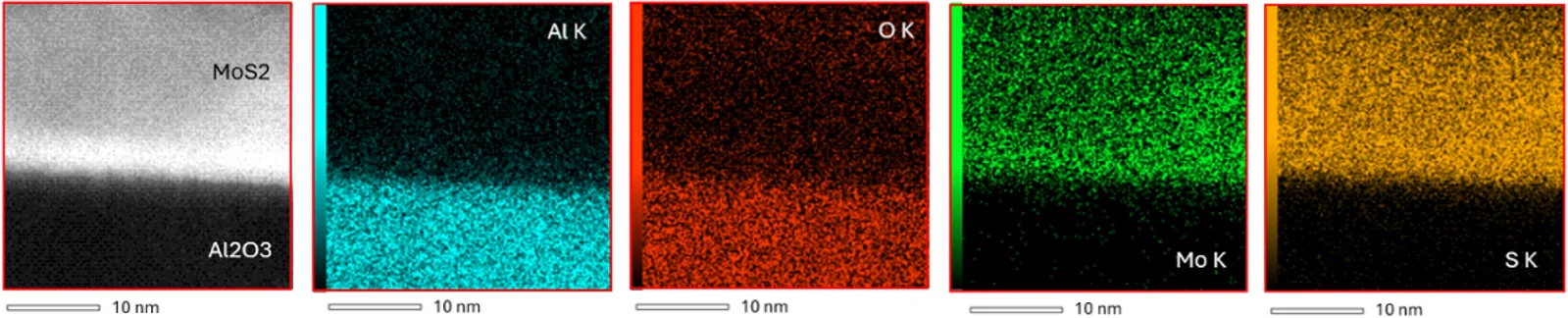
Cross-sectional HAADF-STEM image of the Al_2_O_3_/MoS_2_ interface (leftmost panel), together with corresponding STEM-EDS elemental maps acquired from the same region (right panels). The chemical maps display the spatial distribution of Al K, O K, Mo K, and S K signals, respectively.

**Figure 6:**
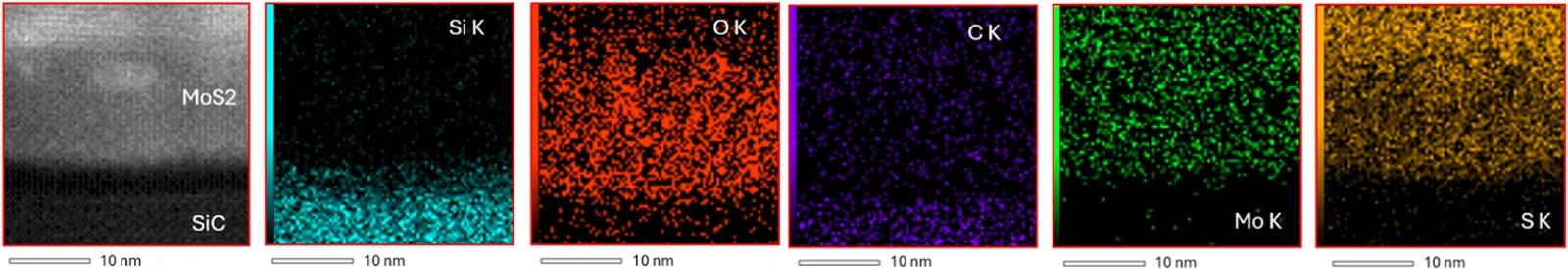
Cross-sectional HAADF-STEM image of the SiC/MoS_2_ interface (leftmost panel), together with corresponding STEM-EDS elemental maps acquired from the same region. The chemical maps display the spatial distribution of Si K, O K, C K, Mo K, S K signals, respectively.

These STEM-EDS findings are fully consistent with the O 1s XPS spectra reported in Figure S3. No clear evidence of significant oxidation reactions at the interface is observed in any of the three systems. The O 1s XPS spectrum of the Al_2_O_3_/MoS_2_ sample exhibits the narrowest and most well-defined line shape, in agreement with the chemical stability of sapphire and with the STEM-EDS evidence of a sharp, well preserved substrate surface.^48^ In contrast, the broader O 1s spectra observed for STO/MoS_2_ and SiC/MoS_2_ reflect more complex oxygen chemical environments. In the STO/MoS_2_ sample, the O 1s features reflect the coexistence of lattice oxygen and lower-coordinated oxygen species which exhibit reduced core-hole screening and therefore appear at higher BE.^49,50^ For the SiC/MoS_2_ sample, the O 1s XPS spectrum shows components associated with oxygen exclusively bonded to Si/C species, in line with the native surface oxidation of the SiC substrate observed by STEM-EDS.^51,52^ Although interfacial interactions between film and substrate may slightly modify the local electronic environment of MoS_2_, the MoS_2_ layer is only 2 ML thick and, thus, the O 1s signal is strongly dominated by the substrate contribution. As a consequence, oxygen directly associated with the buried interface cannot be reliably distinguished from the substrate signal and minor oxygen-related interfacial configurations cannot be uniquely resolved. Therefore, the O 1s spectra alone are not sufficient to unambiguously identify oxygen related interfacial defects. Nevertheless, the Mo 3d spectra (see Figures S1a and b), do not provide evidence for significant Mo oxidation or for the formation of a distinct oxygen-mediated interfacial reaction layer.

Building on these observations, DFT calculations were performed considering different types of defects.^53^ The Density of States (DOS) and the corresponding energy shifts of S 2p core level with respect to the core hole S_0_ in the bulk-like sulfur (E_S0_=-117.325 eV) were calculated and all reported in the Supporting Information. Different sulfur defects (Table S1), the effect of strain (Table S2) and Ti, Sr, Al, C and O substitutions (Table S3) were evaluated. Together with the STEM-EDS measurements, these results provide a basis for explaining the behavior of the S 2p XPS spectra with different substrates shown in Figure 3. In the S 2p fit, the A component is associated with S_0_. The B component appears at higher BE, therefore defects associated with a positive chemical shift are the only considered for the interpretation of our results. We are not able to determine whether there is indeed an effect of strain, because the DFT calculations indicate that this would result in very small shifts, falling well within the experimental uncertainty and the typical error associated with the multicomponent fitting of the XPS S 2p core level. In Figure 7a the core-level energy shifts of the selected defects, with respect to S_0_ bulk-like reference, are reported. These defects correspond, in our assessment, to the most plausible configurations expected at the interface, consistent with the indications provided by STEM-EDS observations. The comparison between the corresponding DFT shifts and the difference between B and A components, is highlighted using a histogram in Figure 7 b.

**Figure 7:**
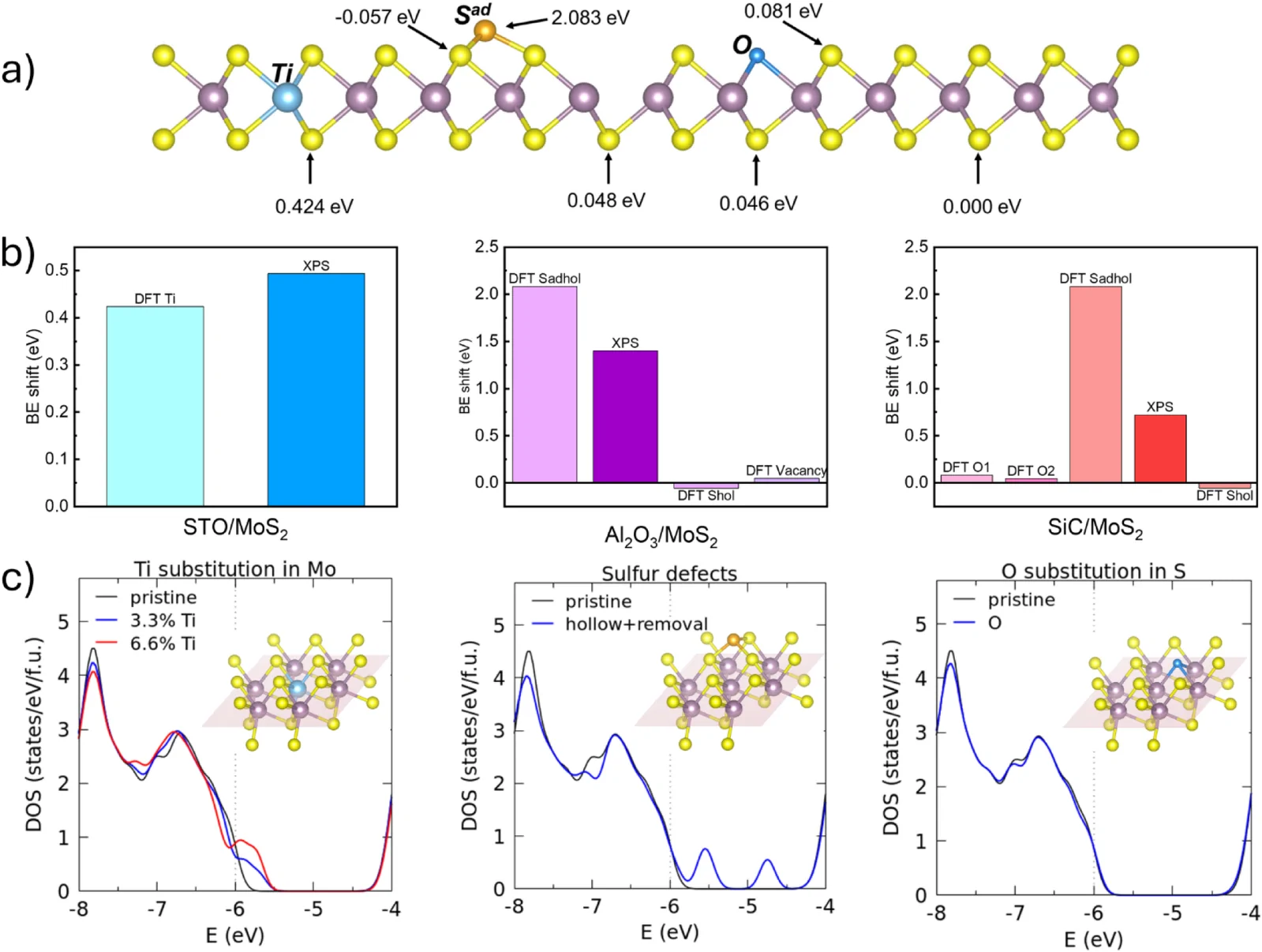
(a) The core-level energy shifts of S 2p states with respect to the bulk-like sulfur used as a reference. b) Histogram of the XPS energy shifts of the B doublets used for the fit (in darker colour blue for STO, violet for Al_2_O_3_ and red for SiC subtrate), together with DFT results (in lighter colours) for selected types of defects. c) DOS calculations for Ti interdiffusion (left panel), S in hollow position (center panel) and O interdiffusion (right panel).

In the case of STO/MoS_2_ STEM-EDS data demonstrate Ti interdiffusion at the interface between film and substrate. Although this technique does not provide direct atomic-scale proof of substitutional Ti incorporation, among the defect configurations considered in our DFT calculations, Ti was modeled as a substitutional impurity occupying a Mo site in the MoS_2_ lattice, as described in the Methods section and shown in Figure S4 in the Supporting Information. This configuration was selected because the observed Ti interdiffusion, together with the similar ionic radii of Ti and Mo, makes substitution at the Mo site the most plausible first-order microscopic model. Among the configurations investigated, this scenario provides the most consistent explanation for the B component observed in the XPS S 2p spectrum. The agreement between the DFT calculation and the XPS fit is quite good, as shown in Figure 7b. However, we cannot rule out the presence of additional sulfur-related defects, which are difficult to discriminate from STEM-EDS measurements. Since the sample grown on Al_2_O_3_ exhibits very sharp interfaces and there is no evidence from the STEM-EDS measurements of any Al or O interdiffusion from the substrate into the MoS_2_, the B doublet can be attributed first to sulfur vacancies, as also indicated by the S/Mo ratio measured by XPS (Figure 2c) of 0.048 eV, which is, however, very small and cannot by itself account for the B doublet. Therefore, we should consider the presence of additional sulfur-related defects. One possible type of defect can be the S adatom at the hollow site S^ad^_hol_ which gives rise to a DFT shift of 2.083 eV. This defect is accompanied by the shift of −0.057 related to the underlying S atom at the hollow site, which however is very small and not considered in our fit of Figure 3b. In this case the agreement between the DFT calculations and the XPS fit is less accurate. However, some differences between DFT and XPS are understandably expected, since DFT calculations have been performed on an ideal monolayer, while XPS measurements have been performed on a 2 ML film grown on single crystal substrates introducing interface effects. In the case of the SiC/MoS_2_ sample, considering the native oxygen observed in the STEM/EDS images (Figure 6), the oxygen defects substituting sulfur atoms were taken into consideration. DFT calculations show that these kind of defects produce higher energy shifts, but very small. Their shifts are 0.081 and 0.046 eV for the two sulfur atoms that are first neighbors to the oxygen defect. In contrast, oxygen adsorbed on top of a sulfur atom would induce a negative energy shift of −2.097 eV (see Supporting Information) which is not compatible with the spectral shape shown in Figure 3c. However, since the B doublet is very broad, oxygen substituted in the sulfur site cannot be the only relevant defect. In addition, oxidation processes were not detected on the basis of the O 1s spectra shown in Figure S3. Moreover, sulfur induced interfacial reactions during the growth process could, in principle, be considered as a possible origin of the B doublet. However, STEM-EDS measurements do not reveal the formation of a distinct sulfur-rich interfacial reaction layer, nor any significant sulfur diffusion into the SiC substrate. Therefore, the available experimental evidence does not allow us to attribute the broad B component to a specific sulfur induced interfacial reaction. Other defects, such as carbon substituting sulfur, which yields a shift of 0.144 eV, sulfur-related defects like S^ad^_hol_ or strain effects should also be considered, which are very likely convoluted with a large amount of structural and morphological disorder, as evidenced by the very broad shape of the S 2p spectrum.

The total DOS calculated by DFT shown in Figure 7c, when compared with the experimental VB spectra of Figure 2d, leads us to conclude that the most likely defect scenario is the occurrence of Ti substitution in STO/MoS_2_, S adatoms at hollow sites in Al_2_O_3_/MoS_2_ and O diffusion in a very disordered SiC/MoS_2_ system. This scenario is also in agreement with the transport measurements shown in Figure 1. We note that the present transport measurements do not provide direct identification of specific point defects, but rather support the substrate dependent interfacial scenarios inferred from XPS, STEM-EDS and DFT results. Within this overall picture, Ti substitution in STO/MoS_2_ is adopted in our DFT modeling as the most plausible microscopic configuration, motivated by the Ti interdiffusion revealed by STEM-EDS and by the close compatibility between the ionic radii of Ti and Mo, which makes substitution at the Mo site energetically plausible. As shown in Figure 7c this defect model introduces additional electronic states close to the VB edge, increasing the DOS at the E_F_ and shifting the VB edge upward, in agreement with the enhanced VB spectral weight observed experimentally (Figures 2d and S2). This is consistent with a p-type electronic character that could explain the metallic-like behavior. The p-type interpretation is proposed as a physically plausible and literature supported interpretation consistent with all our experimental observations and theoretical calculations, rather than direct experimental measurement of p-type conductivity. Ti-related interfacial modifications, which may include substitutional Ti configurations, Ti-induced interfacial states, and/or charge transfer across the interface, can contribute to driving the metallic-like behavior observed in STO/MoS_2_. To the best of our knowledge, experimental evidence for Ti substitution in MoS_2_ or in other TMDCs leading to a well-established p-type doping behavior has not previously been reported in the literature. However, p-type doping in MoS_2_ has been obtained by other substitutional transition metal dopants, as V, Nb or Ta, despite the intrinsic tendency of MoS_2_ toward n-type transport driven by sulfur vacancies.^54–56^ However, Ti cations in STO are quite mobile and can diffuse through STO at the growth temperatures typically used for MoS_2_. When Ti ions migrate toward the surface in sulfur-rich environment, they are energetically favored to form Ti–S bonds. The Ti incorporation into the MoS_2_ lattice is facilitated by the close compatibility between the ionic radii of Ti and Mo, which allows Ti to substitute Mo in either octahedral or trigonal-prismatic coordination with S.^57^ We also note that Ti-induced metallicity has been reported in other semiconductor systems.^58^ In heavily Ti-doped silicon, an insulator-to-metal transition has been experimentally observed when the Ti concentration reaches approximately 10^20^ cm^–3^. This behavior has been interpreted in terms of the formation of a Ti-derived impurity band that overlaps with the Si conduction band, giving rise to delocalized electronic states and metallic transport. Although such concentrations are commonly referred to as hyperdoping in the semiconductor community, they correspond to only ∼0.2 at. % of the Si lattice sites. From the perspective of atomic-scale compositional analysis, this is an extremely small amount of Ti, close to the practical detection limit of STEM-EDS. Consequently, the clear observation of Ti enrichment at the STO/MoS_2_ interface suggests that the local Ti concentration may reach values capable of substantially modifying the electronic structure of the MoS_2_ film and promoting metallic behavior.

To rationalize the emergence of the Ti-induced states near the valence-band edge, we note that Ti^4+^ and Mo^4+^, although chemically equivalent, differ substantially in their electronic configurations. Ti^4+^ is a d^0^ cation, whereas Mo^4+^ is d^2^, with a fully occupied dz^2^-derived state stabilized by the trigonal-prismatic crystal field. Substituting Mo with Ti therefore reduces the local d-electron count on neighboring Mo sites. The lower electronegativity of Ti reduces the covalent character of Ti–S bonding with respect to Mo–S. DFT structural relaxation shows a slight elongation of the Ti–S bond and a redistribution of bonding charge (Figure S7), consistent with the appearance of Mo-dz^2^ states near the Ti impurity site and with the calculated position of the Fermi level slightly below the modified valence band edge, consistent with an effective hole-doping character. These results demonstrate that Ti substitution induces both structural distortions and electronic redistribution, jointly contributing to the emergence of new states near the valence-band edge. Further details are reported in the Supporting Information.

In Al_2_O_3_/MoS_2_, despite the high stability of the Al_2_O_3_ substrate surface, the formation of sulfur defects and vacancies can be related to the highly oxygen terminated and strongly ionic surface of Al_2_O_3_, which can modify the local charge distribution, thus promoting sulfur depletion from the interface. The sulfur defects and vacancies introduce localized states near the Fermi level shown in the calculated DOS of Figure 7c. These localized states hinder carrier mobility and suppress band-like transport, resulting in resistivity that varies only weakly with temperature, as shown in Figure 1.

In SiC/MoS_2_, O substitution does not shift the Fermi level, nor does it introduce localized defect states within the gap as indicated in the calculated DOS of Figure 7c and, moreover, no oxidation of MoS_2_ was detected from the O 1s spectra in Figure S3 and from the Mo 3d spectra in Figure S1. However, as discussed above, other defects, such as carbon substituting sulfur, sulfur related defects and strain-induced distortions, are also plausible and likely coexist at the interface, making the SiC/MoS_2_ interface intrinsically multi-defect and chemically/structurally heterogeneous. We can therefore conclude that all these contributions can act together and are consistent with transport limited by a disordered interfacial environment rather than by a single well-defined substitutional defect. This interpretation is consistent with the XPS Mo 3d fits reported in Figure S1a of the Supporting Information, which show a larger defective component for SiC compared to the other substrates. This can be attributed to the presence of a thin native oxide layer on the SiC surface, which increases the structural and chemical heterogeneity of the interface and thus hampers the formation of an ordered MoS_2_ lattice. In addition, the lattice mismatch between MoS_2_ and SiC is substantially large, leading to significant structural disorder.^15^ The combination of a structurally heterogeneous and highly mismatched interface prevents ordered growth of MoS_2_ on SiC. The fact that neither Arrhenius, Mott variable-range hopping, nor Efros–Shklovskii hopping models provide a consistent description of the resistivity of Figure 1 over a significantly large temperature range, indicates that charge transport in the SiC/MoS_2_ sample cannot be explained by a single microscopic mechanism. Rather, the electrical transport is governed by a distribution of localized states arising from chemical heterogeneity (e.g., native oxide and mixed bonding configurations) together with structural disorder (e.g., lattice mismatch and interface-related defects). Such a disorder-dominated transport regime is fully consistent with the STEM-EDS, XPS and DFT results.

## 3 Conclusions

By examining the interplay of chemical environment and defect formation, both experimentally by XPS and STEM, and theoretically by DFT, we can interpret the temperature dependent behavior of the resistivity of MoS_2_ films grown on STO, Al_2_O_3_ and SiC substrates. For MoS_2_ films grown on STO, the low-resistivity metallic-like behavior can be associated with Ti-related interfacial modifications, with substitutional Ti incorporation representing one of the most plausible microscopic configuration identified by DFT. This interpretation is further supported by previous reports showing that substitutional transition metal dopants can induce p-type behavior in MoS_2_.^54–56^ However, the p-type Ti-substitution model does not exclude alternative microscopic mechanisms or other Ti-related defects that may coexist and contribute to the observed metallic behavior. In contrast, for Al_2_O_3_ and SiC substrates, the transport behavior can be associated with sulfur-related defects and increased chemical/structural disorder, respectively. As reported in the literature, Zhu et al. observed localized trap states arising from structural defects in MoS_2_ films, which tend to reduce mobility.^59^ However, according to a study by Kim et al. on monolayer MoS_2_ grown under different sulfurization conditions, sulfur vacancies introduce shallow donor defects which, by donating electrons to the conduction band, screen the potential of structural defects and thereby enhance electron mobility.^60^ In our case, sulfur vacancies are more abundant in films grown on Al_2_O_3_, whereas we assume that a high degree of structural and chemical disorder is present in the films grown on SiC. These findings are in agreement with the resistivity behavior, which increases strongly with decreasing temperature in the case of SiC, while the resistivity slope is smaller for the film on Al_2_O_3_, even though its room-temperature resistivity is higher than that of the SiC case.

The advanced and complementary techniques employed in this study enabled us to show that unexpected defects can form at the interface even with highly stable substrates. The identified defect configurations should not be regarded as a definitive atomic-scale determination, but they do represent the most consistent reconstruction supported by the experimental and theoretical evidence. On one side, overlooking these interfacial defects may compromise the interpretation of experimental results, on the other side, understanding and controlling them open the possibility to tune interface properties. Considering the central role of the interface between film and substrate, not only for MoS_2_, but also for other TMDCs and, more broadly, for technologically relevant thin film materials, our study provides insights into how interfacial phenomena govern the emergence of new electronic states, which can be used to induce functionalities tailored to specific device applications.

## 4 Methods

### 4.1 Sample Deposition

MoS_2_ thin films were grown by PLD using a KrF excimer laser (λ = 248 nm) at the NFFA APE beamline, Trieste.^61^ To ensure optimal film quality, the growth temperature, background pressure and deposition rate were systematically optimized by varying the PLD parameters and monitoring the resulting XPS spectra, until the characteristic spectral features of MoS_2_ were consistently obtained. The laser beam was focused onto a stoichiometric polycrystalline MoS_2_ target (99.99% purity) with a fluence of approximately 2 J/cm^2^. The target-to-substrate distance was fixed at 5 cm. Depositions were carried out at a substrate temperature of 650 °C on three different single-crystal substrates: SrTiO_3_ (111), c-axis Al_2_O_3_ (0001), and 6H-SiC (0001). No specific surface treatment, chemical etching, or surface reconstruction procedure was performed prior to growth. The substrates were intentionally used in their commercially supplied condition in order to investigate MoS_2_ growth on technologically relevant and readily available functional substrates. The films were deposited under ultra-high vacuum (UHV) conditions, with a base pressure of about 10^–8^ mbar and without intentional gas introduction during growth. The low residual gas pressure minimizes contamination from atmospheric species and suppresses unintentional oxygen incorporation from the growth environment. Similar growth conditions have previously been employed for oxygen-sensitive chalcogenide materials, such as Bi_2_Se_3_, FeSe and Cr_4_Te_5_, without evidence of oxygen contamination.^62–64^ The growth rate was calibrated by X-ray reflectivity measurements and was determined to be ∼200 laser pulses per unit cell, corresponding to one monolayer of MoS_2_. After deposition, the samples were cooled to room temperature under the same pressure conditions as those used during growth.

### 4.2 Transport Measurements

Electrical transport measurements were performed by four probe (van der Paw) method using a Janis Closed-cycle cryostat over the temperature range T = 10 K–325 K. The ramp for the electrical transport is around 3 K/min.

### 4.3 Photoelectron Spectroscopy

XPS measurements were performed by synchrotron radiation after *in situ* transferring from the PLD system directly connected to the transfer chamber of the APE-HE beamline at Elettra synchrotron in Trieste. The measurements were performed at room temperature using a hemispherical electrostatic analyzer with the sample at 45° with respect to the impinging linearly polarized light and normal to the surface. The binding energies of the photoemission peaks were calibrated with respect to the Fermi level of Au reference samples. The excitation energies were set at 1110 eV for survey scans and core levels and 540 eV for VB spectra. The overall energy resolution (photon bandpass and electron energy analyzer Omicron EA125) was set to 300 meV.^65^

### 4.4 Transmission Electron Microscopy

HAADF-STEM imaging and STEM-EDS mapping were performed on a JEOL F200 TEM/STEM operated at 200 kV and equipped with a cold field-emission gun (cold FEG) and with a dual-detector EDS system. Imaging was carried out in STEM mode using a convergence semi-angle of 20 mrad, yielding a probe diameter of approximately 0.16 nm, while HAADF signals were collected over an angular range of 20–73 mrad.

Cross-section TEM lamellae were prepared from all three samples using a TESCAN Amber X plasma FIB-SEM via a site-specific lift-out procedure. The extracted lamellae were subsequently thinned and polished to electron transparency to enable STEM analysis of the film/substrate interface.

### 4.5 Computational Details

We performed first-principles calculations within the framework of Density Functional Theory (DFT) as implemented in the Quantum ESPRESSO suite.^66^ Norm-conserving pseudopotentials of the Trouiller-Martins type^67^ have been adopted, while the Perdew–Burke–Ernzerhof (PBE) functional^68^ has been used for the exchange-correlation energy within the generalized gradient approximation. We adopted a Gaussian smearing with a width of 0.01 Ry and a plane-wave cutoff of 100 Ry. Starting from bulk 2H-MoS_2_ with in-plane lattice parameter of 3.16 Å,^69^ we constructed 15.80 × 16.42 Å supercells for simulating a single MoS_2_ layer, with 25 Å of vacuum separating periodic copies along the direction z perpendicular to the monolayer plane and adopting the truncation scheme for Coulomb interaction in the z direction.^70^ A 3×3×1 mesh using the Monkhorst–Pack scheme^71^ has been used for sampling the Brillouin zone, and the atomic positions have been optimized in all structures until forces were smaller than 10^–3^ Ry bohr^–1^. Core-level binding energies of 2p states of S atoms have been calculated using the core-excited pseudopotential method.^72^ To simulate photo-excited ions, we used the code atomic of the Quantum ESPRESSO suite to generate a norm-conserving pseudopotential that includes a hole in the 2p subshell of sulfur. A scalar-relativistic approach has been adopted, implying that calculated core-level binding energies correspond to an average binding energy missing the splitting arising from spin-orbit coupling. The core-excited pseudopotential has been tested against known core-electron binding energies of simple molecules tabulated in Ref.,^73^ yielding an overall agreement with errors below 10%.

## Supplementary Material



## Notes

Description of X-ray photoemission fitting and analysis, including Mo 3d and O 1s core levels and valence band spectra. Details of Density Functional Theory calculations, including S 2p core level shifts and projected density of states for different defect configurations. Effect of Ti substitution in MoS_2_, including band structures, projected density of states and partial charge density. Figures S1–S7 and Tables S1–S3 (PDF)
