# Supplementary material for: Interfacial Properties of MoS2 Thin Films Grown on Functional Substrates
Source: ACS Omega. 2026 Sep 7;11(37):55454–64. doi: 10.1021/acsomega.6c03932 (PMC13613928; doi:10.1021/acsomega.6c03932)
Supplement: Supplementary file 1 [file ao6c03932_si_001.pdf]

Supporting Information

Interfacial properties of MoS_2 thin films grown on functional substrates

Hafiz Sami Ur Rehman[1], Nunzia Coppola[1], Alice Galdi[1], Sandeep Kumar Chaluvadi[2], Shyni Punathum Chalil[2], Pasquale Orgiani[2, 3], Sara Passuti[3], Regina Ciancio[3], Paolo Barone[4], Luigi Maritato[1] and Carmela Aruta[4,]*

[1]Dipartimento di Ingegneria Industriale-DIIN, Università Degli Studi di Salerno, Fisciano, Salerno 84084, Italy

[2]CNR-IOM, Strada Statale 14 Km 163.5, Basovizza, Trieste 34149, Italy

[3]Area Science Park, Padriciano 99, Trieste 34149, Italy

[4]CNR-SPIN, Via del Fosso del Cavaliere 100, Roma 00133, Italy

*carmela.aruta@cnr.it

1. X-ray photoemission spectroscopy fitting

We collected X-ray photoemission spectroscopy (XPS) survey scans, Mo 3d and S 2p core levels and valence band (VB) spectra on MoS_2 samples grown on $SrTiO_3$ (STO), Al_2O_3 and SiC substrates. Excitation energies of 1110 eV were employed for the survey and core-level measurements, while 540 eV was used for the VB spectra. Binding energy (BE) calibration is performed with reference to the adventitious C 1s at 284.8 eV. For the fitting procedure we used CasaXPS software (Casa Software, Teignmouth, UK).[1,2] A Shirley function is assumed for background subtraction and a multicomponent deconvolution procedure is performed, using mixed Gaussian and Lorentzian line shapes. For the S 2p fitting of Figure 3 of the main manuscript we used two components A and B made by doublets of the $2p_{3/2}$ and $2p_{1/2}$ spin-orbit splitting. We fixed as much as possible the fitting parameters. For all the doublets the splitting is fixed at 1.2 eV, the degeneracy ratio is 1:2 for the spin-orbit area ratio and the FWHM is a free parameter but kept the same for both peaks. The component A represents the stoichiometric sulfur.

The fit of the Mo3d shown in Fig. S1 was performed with two pairs of $3d_{5/2}$ and $3d_{3/2}$ spin-orbit doublets for the Mo^{4+} and a higher binding energy (BE) contribution (defective Mo at higher valence state). A small contribution from the Mo^{6+} at even higher BE is hardly detectable, also owing to the *in situ* transfer of the samples to the XPS analysis chamber, which limits the surface oxidation. In the fitting procedure the spin orbit splitting was fixed at 3.14 eV for all the doublets, the degeneracy ratio was 2:3 for the spin-orbit area ratio and the full width at half maximum (FWHM) was a free parameter but fixed at the same value for the two peaks of each doublet. At about 226.5 eV the Mo-

S2s component is also included in the fit. In panel (a) the results for the MoS_2 films on different substrates (STO, Al_2O_3 and SiC) are reported, while in panel (b) the comparison between different thicknesses (1ML, 2ML and 3ML) of the MoS_2 films grown on STO is reported, as this system provides the most well defined and least disordered interface among the investigated substrates, allowing the evolution of the spectral components with thickness to be more clearly resolved. This thickness dependent study shows that the higher binding energy doublet is associated with the interface between film and substrate, as it tends to decrease as the thickness increases.

The stoichiometry of the samples (Figure 2c of the main manuscript) is obtained from the S^{2-}/Mo^{4+} total area ratio in survey scans, with Scofield photoionization cross-sections, rescaled as relative sensitivity factors (RSF) in CasaXPS by correction for the energy-dependent transmission of the analyzer.[3] RSF values used for Mo 3d and S 2p core levels from CasaXPS database were 9.5 and 1.67 respectively. However, at high excitation energies the ratio between the two cross sections changes less than 2%. This is well below the expected quantification uncertainty, arising from instrumental effects (energy-dependent analyzer transmission, detector efficiency and setup geometry), matrix and attenuation effects (local electron density, surface roughness, and chemical composition), uncertainties in the fitting procedure (background choice, number of components, and peak deconvolution), as well as noise, surface charging and energy drift.

The valence band maximum (VBM) is obtained from the fitting of the VB spectra by linear regression in the Mo $4dz^2$ energy region (Fig. S2). The VBM is obtained from the intersection with zero intensity.[4] In the case of STO and Al_2O_3, the defect states calculated by DFT as described in the main text contribute to the shift of the Fermi level towards the valence band, thus decreasing the VBM.

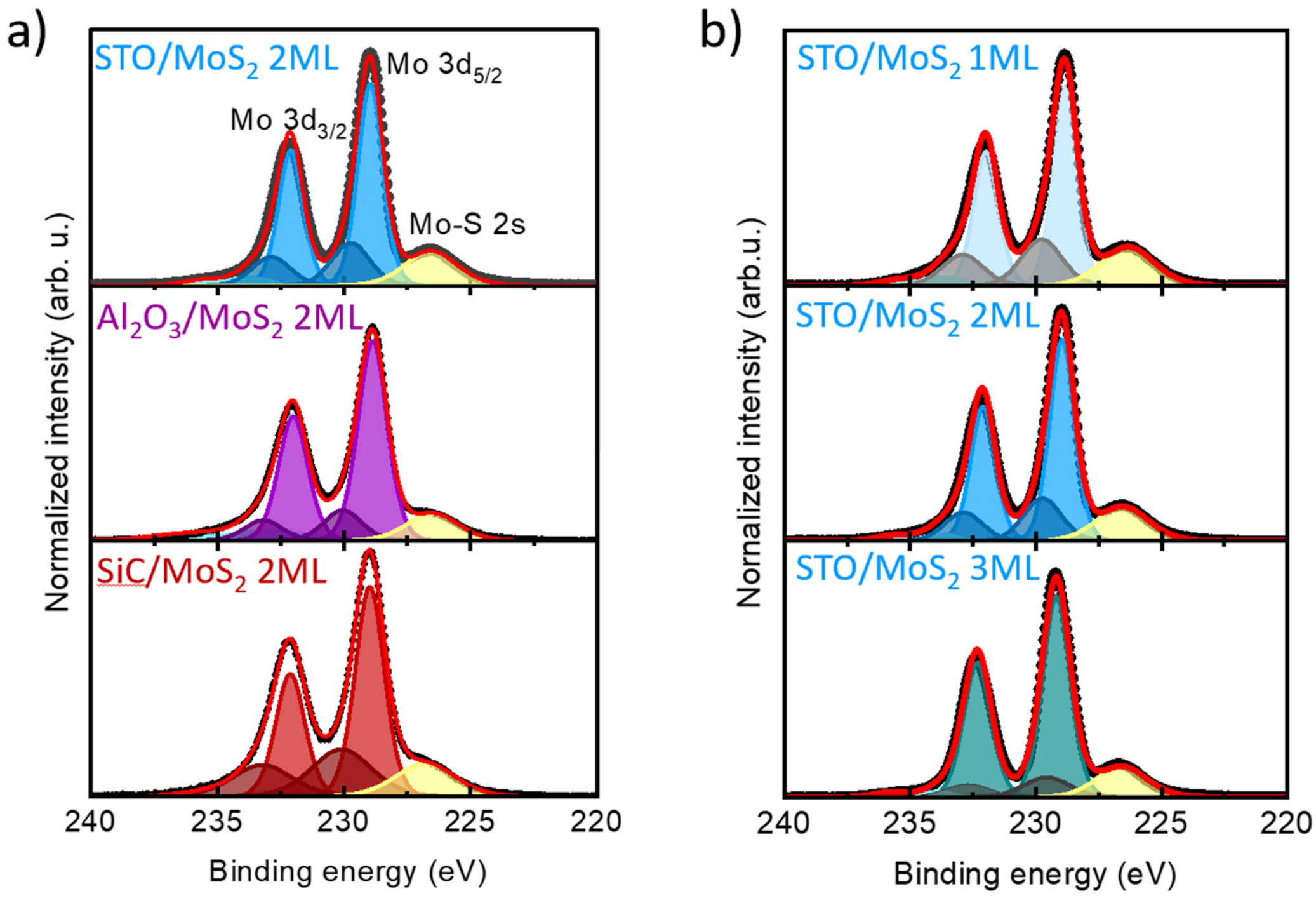

Figure S1. XPS Mo3d core level (a) of MoS_2 on STO, Al_2O_3 and SiC substrates, and (b) of MoS_2 on STO with 1ML, 2ML and 3ML thickness. The multicomponent fit is also shown.

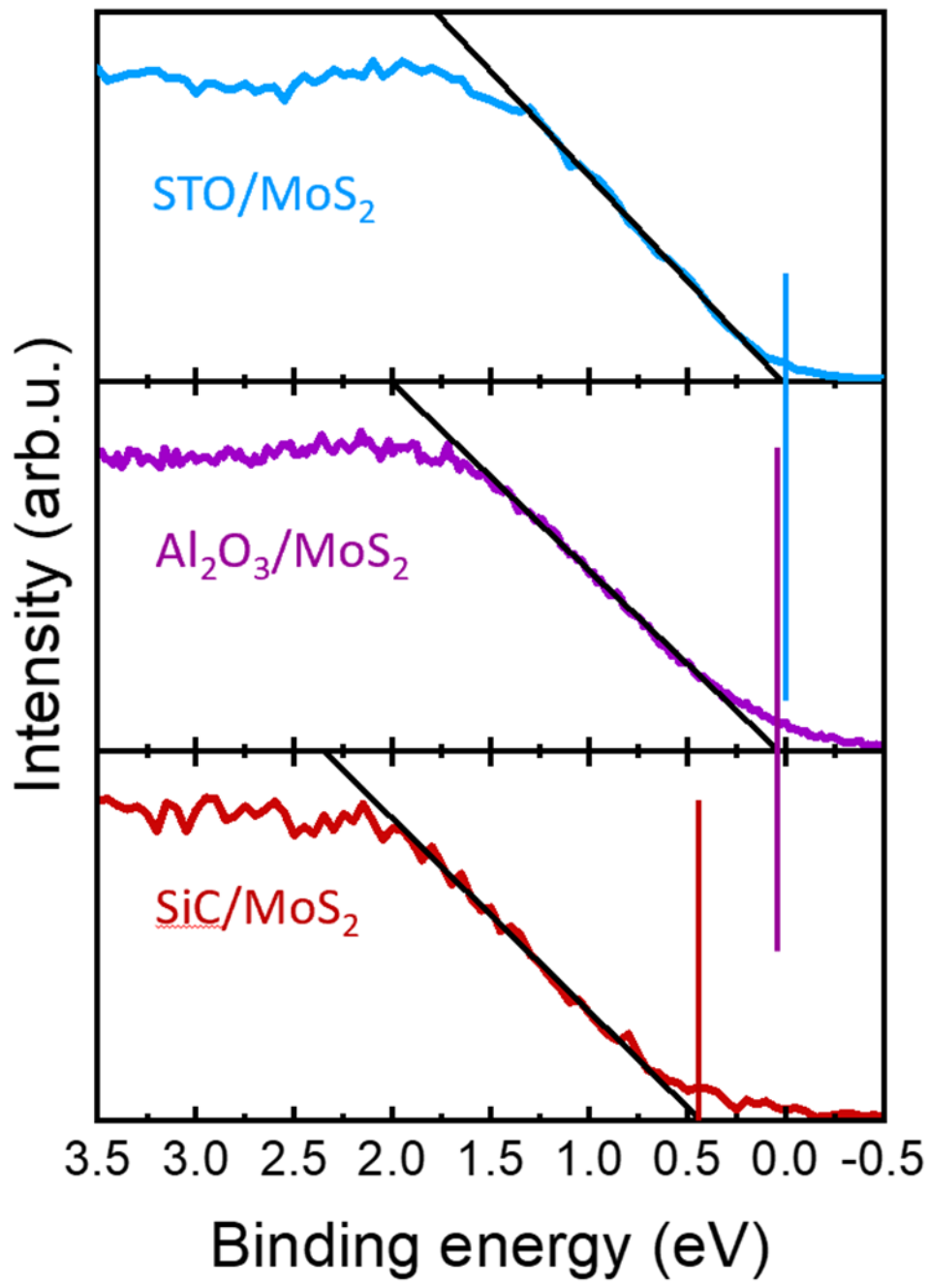

Figure S2. VB spectra close to the Fermi level to highlight the calculated valence band maximum (VBM): +0.420 eV (SiC/MoS_2), +0.026 eV (Al_2O_3/MoS_2) and +0.020 eV (STO/MoS_2).

O1s spectra reported in Fig. S3 show that in case of Al_2O_3/MoS_2 only one component associated to the oxygen in the substrate lattice can be considered.[5] In the case of STO/MoS_2 and SiC/MoS_2 two components have been used for the fit. Concerning STO/MoS_2 they can be assigned to lattice oxygen and lower-coordinated oxygen for the components at lower and higher BE respectively.[6] In the case of SiC/MoS_2 the two components can be associated to O-Si-C and SiO_x at lower and higher BE respectively.[7] In both STO/MoS_2 and SiC/MoS_2, the additional O 1s components are too intense to be attributed solely to a nanometric interfacial layer. They can instead be consistently assigned to substrate-related oxygen environments (native surface oxidation, low-coordination or defect-related oxygen). Therefore, the assignment of the individual components remains ambiguous and due to the overwhelming substrate contribution, the interfacial signal cannot be reliably distinguished.

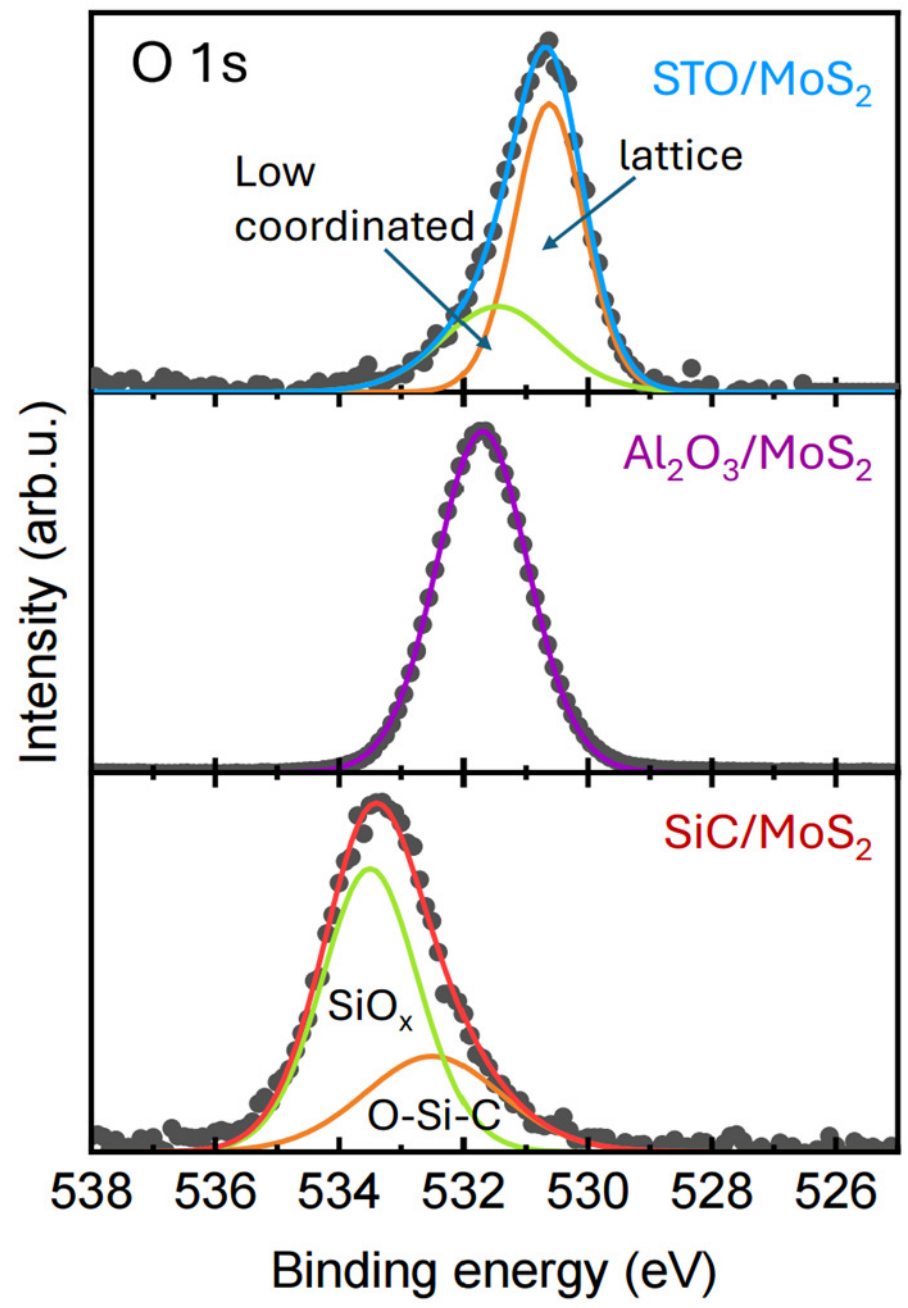

Figure S3. XPS O1s core level extracted from the survey scans of MoS_2 on STO, Al_2O_3 and SiC substrates.

2. Density Functional Theory calculations

Density Functional Theory (DFT) calculations of the S 2p core-level shift for different types of defects are calculated as described in the Methods section of main text. Figure S4 schematically illustrates the 5X3 MoS_2 supercell together with the defect configurations considered in this work. Intrinsic defects (sulphur vacancies and sulphur adatoms) and substrate-related defects (Ti, Sr and Al substituting Mo atoms, and O or C substituting S atoms) were introduced one at time, followed by full structural relaxation. For the STO/MoS_2 interface, Ti was modeled as a substitutional impurity occupying a Mo site. This configuration was selected because STEM-EDS reveals Ti interdiffusion across the interface, while the similar ionic radii of Ti and Mo make substitution at the Mo site the most plausible first-order microscopic model. The calculated S 2p chemical shifts are reported in Tables S1–S3 with respect to sulfur in the bulk-like MoS_2.

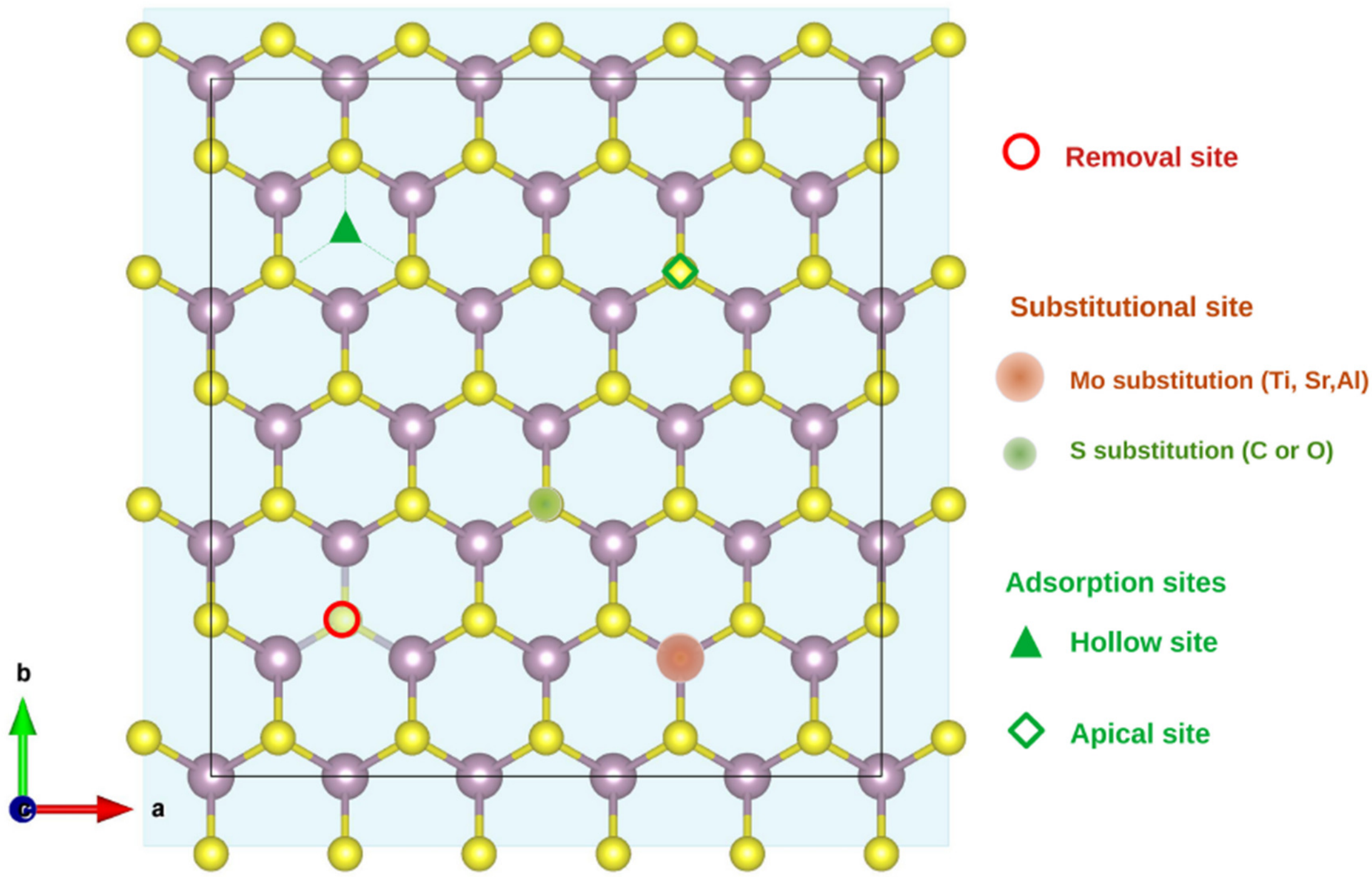

Figure S4. Schematic representation of the 5 × 3 MoS_2 supercell adopted for the DFT calculations and of the defect configurations considered in this work. The same supercell was used to model sulfur vacancies, sulfur adatoms at apical and hollow sites, substitution of Mo atoms by Ti, Sr or Al, and substitution of S atoms by O or C. All structures were fully relaxed before calculating S 2p core-level shifts and density of states.

Chemical shifts (eV) of sulfur defects	S_{rem}	S_{api}	S_{hol}	S_{api}^{ad}	S_{hol}^{ad}
	0.048	-1.507	-0.057	0.870	2.083

Table S1. Calculated core-level energy shifts of sulfur defects with respect to ordinary S in a pristine MoS_2 monolayer. Shifts are calculated for sulfur atoms that are closest to the defect labeled by the subscript. Three kinds of defects have been considered: a sulfur vacancy, a sulfur adsorbed on top of another sulfur and a sulfur adsorbed on the hollow site above the hexagonal MoS_2 lattice. Chemical shifts of adsorbed S defects, denoted by the superscript “ad”, are also listed.

strain	0%	+2%	-2%
**S_{rem}**	0.048	0.026	0.074
**S_{api}**	-1.507	-1.527	-1.488
**S^{ad}_{api}**	0.870	0.919	0.813

Table S2. Calculated core-level energy shifts with different strain conditions for the sulfur adsorbed on top of another sulfur and a sulfur adsorbed on the hollow site above the hexagonal MoS_2 lattice, already reported in Table S1.

S chemical shifts (eV) due to other defects	Ti	Sr	Al	C	O
	0.424	0.766	0.596	0.091/0.045	0.081/0.046

Table S3. Calculated core-level energy shifts of S 2p close to substitutional sites. We considered substitution of Mo atoms with Ti, Sr, Al and of sulfur atoms with C or O. In the latter case, we report two values, corresponding to a hole in a nearest-neighbor sulfur atom belonging respectively to the same S layer where a sulfur atom has been replaced or to the other S layer.

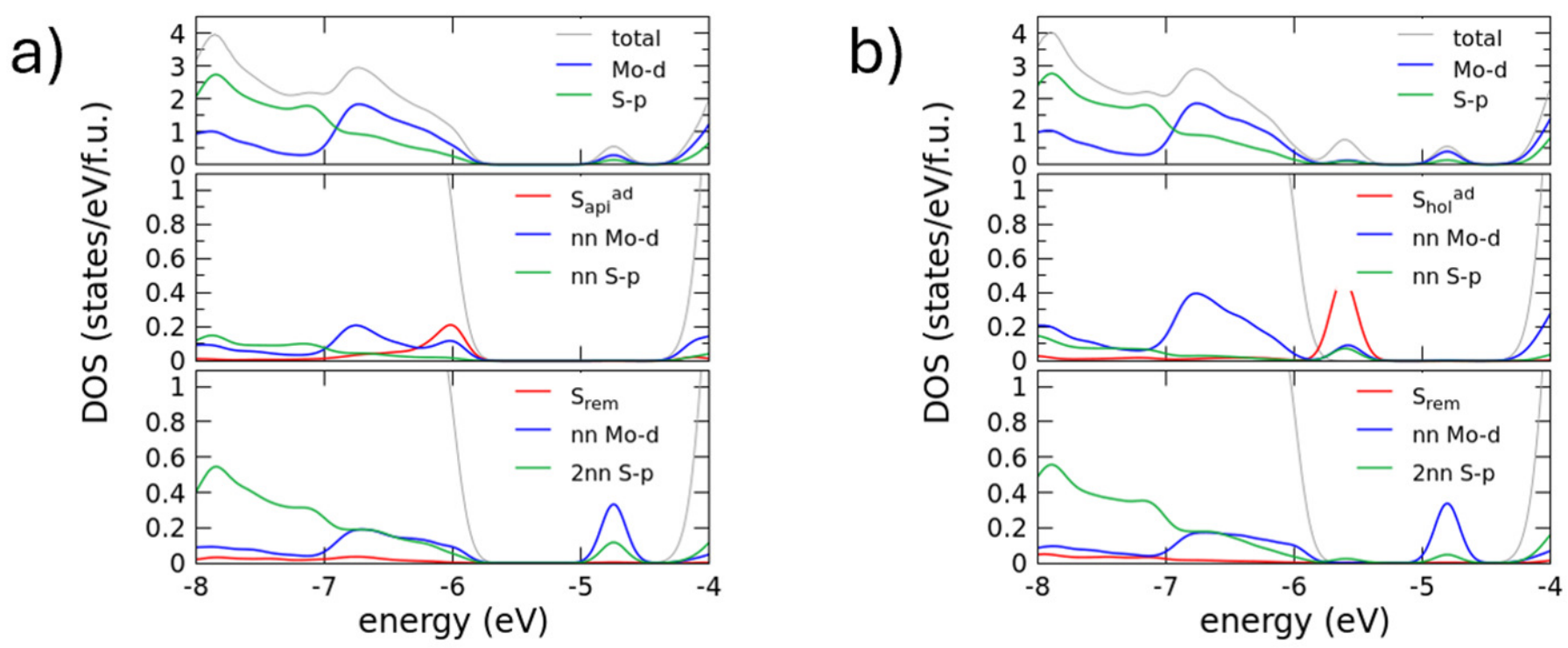

Figure S5. Projected density of states (energy expressed with respect to the vacuum level) of the sulfur defects reported in table S1 . a) sulfur adsorbed on top of another sulfur S_{api}^{ad}, together with the nearest neighbor sulfur vacancy S_{rem}; b) sulfur adsorbed on the hollow site S_{hol}^{ad}, together with the nearest neighbor sulfur vacancy S_{rem}.

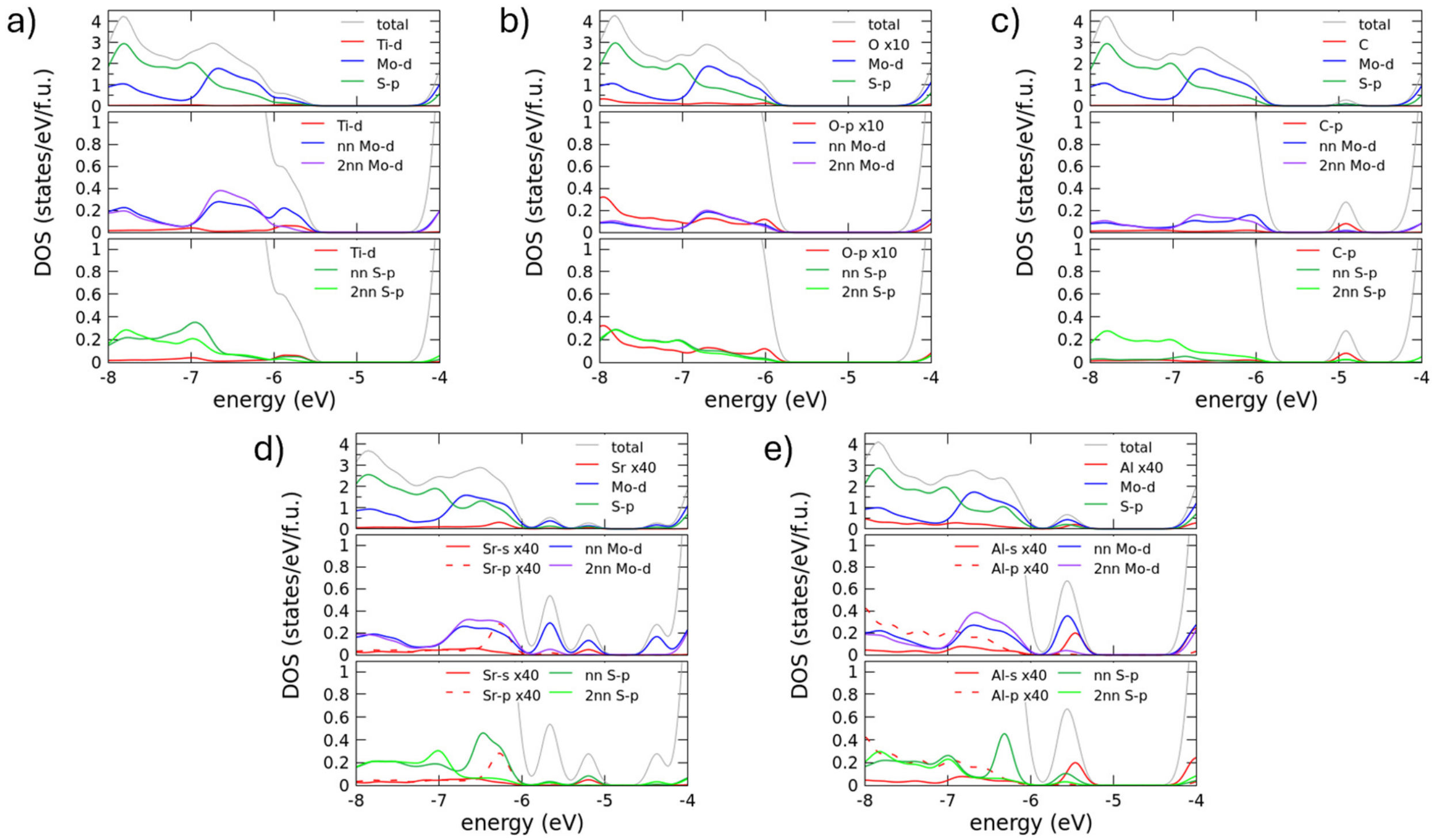

Figure S6. Projected density of states calculated for the substrate-related defect configurations reported in Table S3: a) substitution of Mo atoms with Ti, b) substitution of sulfur atoms with O, c) substitution of sulfur atoms with C, d) substitution of Mo atoms with Sr, e) substitution of Mo atoms with Al. The different substitutional defects produce distinct modifications of the electronic structure close to the valence-band edge. In particular, substitutional Ti introduces additional electronic states in the vicinity of the Fermi level, consistent with the enhanced valence-band spectral weight experimentally observed for STO/MoS_2.

2.1 Effect of Ti substitution in MoS_2

The comparison between the pristine and the Ti substituted MoS_2 is performed with an isolated, freestanding MoS_2 layer (using the same orthorhombic/rectangular supercell comprising 30 units of MoS_2). The band structure of the stoichiometric MoS_2 (Figure S7 a) shows a valence band edge (VBE) dominated by Mo dz^2 (even though a sizeable degree of hybridization appears, as expected). The small tail of the DOS above the VBE is to be ascribed to the Gaussian smearing adopted for Brillouin-zone integration: consistently, partial occupancies are either exactly 1 below the VBE or 0 above it.

Structural relaxation after replacing one Mo atom out of 30 by Ti in the same supercell, reveals both charge redistribution and local structural distortions. It can be observed in Figure S7 c) a depletion of bonding charge around the Ti impurity and a slight elongation of Ti-S bonds, which also causes a small reduction of the neighbouring Mo–S bond lengths. This behaviour reflects the lower electronegativity of of Ti (1.54 in the Pauling scale, compared to 2.16 for Mo and 2.58 for S) which reduces the covalent character of Ti–S bonding. The calculated Ti–S bond length (2.423 Å) is consistent with that of TiS_2, while the Mo–S bond length in pristine MoS_2 (2.408 Å) matches well the experimental value (2.41 Å).[8,9]

The band structure of Ti-substituted MoS_2 (Figure 7b) shows a small positive shift of the valence band edge relative to pristine MoS_2. Additional states with Mo-dz^2 character appear on Mo atoms nearest to the Ti site, while Ti-d states contribute mainly to the conduction manifold. The calculated Fermi level lies slightly below the modified VBE, consistent with an effective hole-doping character.

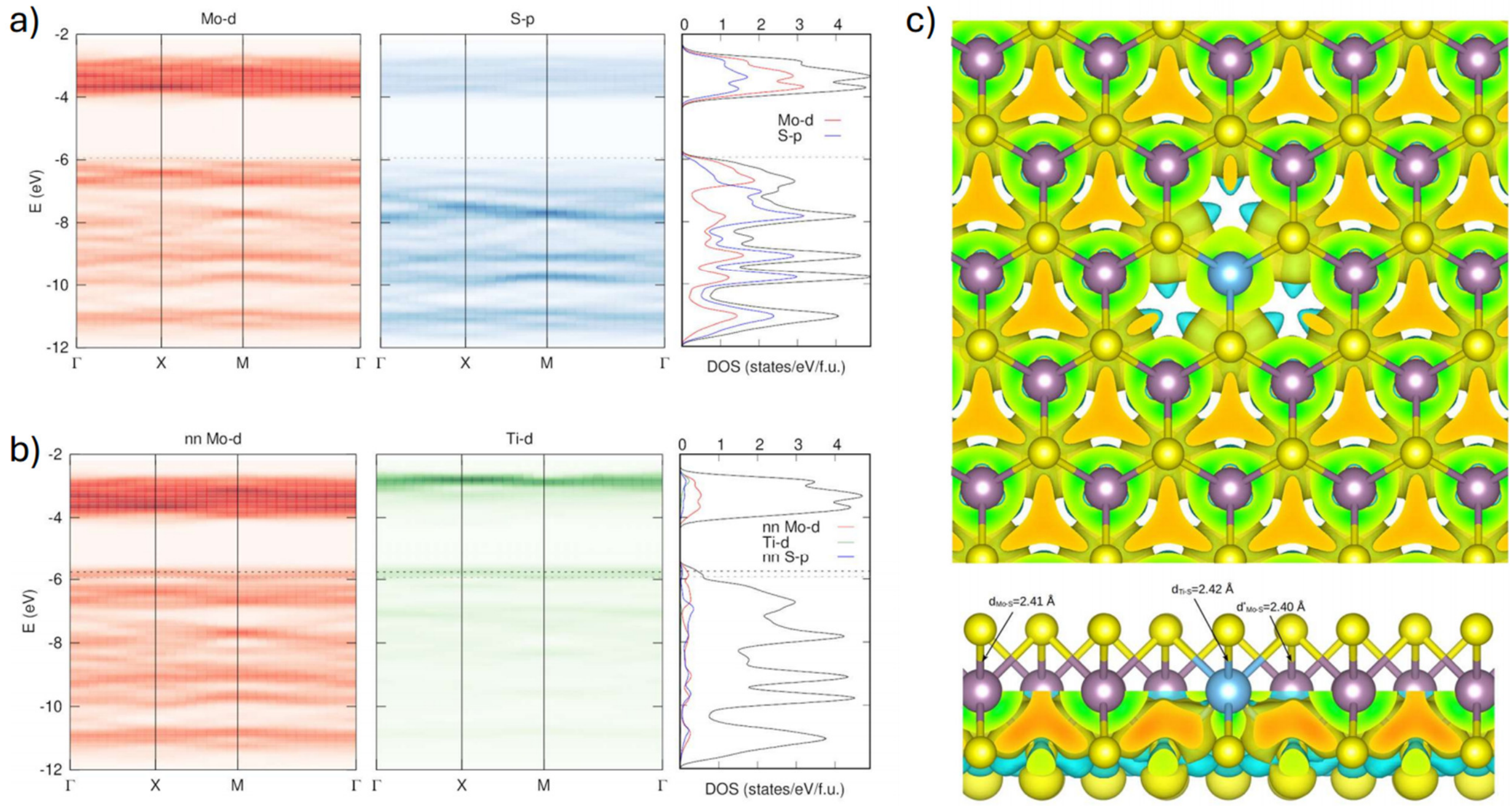

Figure S7. a) Band structure of pristine MoS_2 in the adopted supercell highlighting Mo-d and S-p character, alongside the projected DOS; b) Band structure of Ti-substituted MoS2 in the adopted supercell highlighting the character of Ti-d states and Mo-d and S-p for nearest neighbors. The gray horizontal dashed line is the VBE of pristine MoS2, while the black horizontal dashed line is the calculated Fermi level. c) Partial (bond) charge density, highlighting a depleting of bonding charge around Ti impurity. Ti-S bond is slightly elongated, causing a small reduction of the neighboring Mo-S bond lengths.

References

(1) N. Fairley. CasaXPS: A Software Package for Electron Spectroscopy. *in Surface Analysis by Auger and X-ray Photoelectron Spectroscopy, D. Briggs and J. T. Grant Eds. IM Publications:* **2009**, 441–460.

(2) Neal Fairley. Systematic and Collaborative Approach to Problem Solving Using X-Ray Photoelectron Spectroscopy. *Applied Surface Science Advances* **2021**, *5*, 100112.

(3) J.J. Yeh, I. Lindau. Atomic Data and Nuclear Data Tables. **1985**, *32*, 1–155.

(4) Lin, Y.-K. et al. Thickness-Dependent Binding Energy Shift in Few-Layer MoS2 Grown by Chemical Vapor Deposition. *ACS Applied materials & Interfaces* **2016**, *8*, 22637.

(5) Shiro Ozaki, Yusuke Kumazaki, Naoya Okamoto, Yasuhiro Nakasha, Toshihiro Ohki, Naoki Hara. Effect of Oxidant Sources on Carbon-Related Impurities in ALD-Al2O3 for Solid-State Devices. *Appl. Phys. Express* **2023**, *16*, 091001–091004.

(6) Kelsey A. Stoerzinger, Ryan Comes, Steven R. Spurgeon, Suntharampillai Thevuthasan, Kyuwook Ihm, Ethan J. Crumlin, Scott A. Chambers. Influence of LaFeO3 Surface Termination on Water Reactivity. *J. Phys. Chem. Lett.* **2017**, *8*, 1038–1043.

(7) M.S. Chandrasekar, N.R. Srinivasan. Role of SiOx on the Photoluminescence Properties of β-SiC. *Ceramics International* **2016**, *42*, 8900–8908.

(8) R. R. Chianelli, J. C. Scanlon, A. H. Thompson. Structure Refinement of Stoichiometric TiS2. *Materials Research Bulletin* **1975**, *10*, 1379–1382.

(9) Roscoe G. Dickinson, Linus Paulingl. THE CRYSTAL STRUCTURE OF MOLYBDENITE. *JACS* **1923**, *45*, 1466–1471.
